# A clinical prediction tool for extended-spectrum β-lactamase-producing Enterobacteriaceae urinary tract infection

**DOI:** 10.1186/s12879-022-07040-y

**Published:** 2022-01-13

**Authors:** Hui Liu, Suishan Qiu, Minghao Chen, Jun Lyu, Guangchao Yu, Lianfang Xue

**Affiliations:** 1 Department of Pharmacy, The First Affiliated Hospital of Jinan University, Guangzhou, 510630 Guangdong China; 2 Department of Clinical Research, The First Affiliated Hospital of Jinan University, Guangzhou, 510630 Guangdong China; 3 Department of Laboratory Medicine, The First Affiliated Hospital of Jinan University, Guangzhou, 510630 Guangdong China; 4 Department of Pharmacy, The First Affiliated Hospital of Jinan University, 163# HuangPu Avenu West, Tianhe District, Guangzhou, 510620 China

## Abstract

**Background:**

Prevalence of extended-spectrum beta-lactamase-producing-Enterobacteriaceae (ESBL-E) has risen in patients with urinary tract infections. The objective of this study was to determine explore the risk factors of ESBL-E infection in hospitalized patients and establish a predictive model.

**Methods:**

This retrospective study included all patients with an Enterobacteriaceae-positive urine sample at the first affiliated hospital of Jinan university from January 2018 to December 2019. Antimicrobial susceptibility patterns of ESBL-E were analyzed, and multivariate analysis of related factors was performed. From these, a nomogram was established to predict the possibility of ESBL-E infection. Simultaneously, susceptibility testing of a broad array of carbapenem antibiotics was performed on ESBL-E cultures to explore possible alternative treatment options.

**Results:**

Of the total 874 patients with urinary tract infections (UTIs), 272 (31.1%) were ESBL-E positive. In the predictive analysis, five variables were identified as independent risk factors for ESBL-E infection: male gender (OR = 1.607, 95% CI 1.066–2.416), older age (OR = 4.100, 95% CI 1.678–12.343), a hospital stay in preceding 3 months (OR = 1.872, 95% CI 1.141–3.067), invasive urological procedure (OR = 1.810, 95% CI 1.197–2.729), and antibiotic use within the previous 3 months (OR = 1.833, 95% CI 1.055–3.188). In multivariate analysis, the data set was divided into a training set of 611 patients and a validation set of 263 patients The model developed to predict ESBL-E infection was effective, with the AuROC of 0.650 (95% CI 0.577–0.725). Among the antibiotics tested, several showed very high effectiveness against ESBL-E: amikacin (85.7%), carbapenems (83.8%), tigecycline (97.1%) and polymyxin (98.2%).

**Conclusions:**

The nomogram is useful for estimating a UTI patient’s likelihood of infection with ESBL-E. It could improve clinical decision making and enable more efficient empirical treatment. Empirical treatment may be informed by the results of the antibiotic susceptibility testing.

## Background

Extended-spectrum beta-lactamase producing Enterobacteriaceae (ESBL-E) are a diverse family of Gram-negative bacteria, mainly *Escherichia coli (E. coli)* and *Klebsiella pneumoniae (K. pneumoniae),* which express a clinically concerning drug resistance mechanism [1]. ESBL-E can hydrolyze and eliminate most broad-spectrum β-lactam antibiotics. Compared with non-ESBL-E infections, serious infections caused by ESBL-E have higher morbidity and mortality [2].

ESBL-E hydrolysis of carbapenem antibiotics is low, so carbapenem antibiotics are often used as the first choice in clinical treatment of ESBL-E infections. However, the abuse of carbapenems may result in the selection of carbapenem resistant Enterobacteriaceae, which will ultimately make it more difficult to treat this kind of bacteria [3, 4].

UTIs are the most common class of infectious disease, and antibiotics are their main means of treatment. The most common pathogen group in urine cultures is PE, which accounts for 30% to 40% of all urine culture bacteria [5]. In recent years, ESBL-E infection has been on the rise, and it is the main cause of hospital and community-acquired infections. In a study of antimicrobial resistance trends from 2010 to 2013, ESBL-E was frequently detected in China and Southeast Asia, and the ESBL production rate of *E. coli* and *K. pneumoniae* in some Asian countries was as high as 60% [6]. A study by Vachvanichsanong estimated that ESBL-E represented one-third of all *E. coli* and *K. pneumoniae* UTI episodes [7]. Data from the CHINET antimicrobial resistance monitoring project shows that the ESBL-producing isolates resistant to 3rd generation of cephalosporins increasing from 52.2 to 63.2% between 2005 and 2014 in China [8].

Several studies have suggested that infections caused by ESBL-E have an important clinical impact, and the growing prevalence of these microorganisms in hospitals had been well proven [2, 4]. Urinary tract infections (UTIs) are the main type of bacterial infection in hospitalized patients, and many of these exhibit resistances to the first-line antibiotics usually used to treat UTIs. Infections caused by ESBL-E frequency increased at a faster rate in health associated settings than in the community between 2014 and 2020 [9]. Patients who are identified to be at risk of ESBL-E infection can have their treatment empirically tailored to reduce treatment failure, complications, and antibiotic costs, and to avoid improper use of carbapenem drugs, reducing the risk of selecting drug-resistant microorganisms [10].

A key component of managing ESBL-E infection is to predict its incidence. A highly accurate predictive model can help identify high-risk patients and prevent or reduce the incidence of ESBL-E infection. However, neither test indicators nor imaging tests can yet predict ESBL-E infection. Therefore, this study aims to determine the prevalence and risk factors of ESBL-E infection in hospitalized patients with urinary tract infections and to establish a reliable predictive model.

## Methods

### Study population

This study was conducted at a university-affiliated tertiary hospital with 1900 beds. This study was conducted at the first affiliated hospital of Jinan university, a university-affiliated tertiary hospital in Guangzhou, China with 1900 beds. All cases from January 2018 to December 2019 in which all of a patient’s urine cultures tested positive for Enterobacteriaceae were reviewed. All non-repetitive mid-stream urine (MSU) samples obtained during the study period with a positive urine culture of either *E. coli* or *K. pneumoniae* were included in the analysis. In the annual microbiological epidemiology report of our hospital, 95% of the urine bacterial culture results were *E coli* and *K. pneumoniae,* and *Proteus mirabilis* and *Salmonella* accounted for the remaining 5%. However, no strains of ESBL positive were found in the strains of *Proteus mirabilis* and *Salmonella*, so these two types of bacteria were not included in this study. UTIs were defined in accordance with uniform diagnostic criteria of the European Society of Clinical Microbiology and Infectious Diseases (ESCMID) [11]. Patients were excluded from the study if their medical records were missing data or if one or more of their samples were multi-microorganismal-defined as containing two or more pathogenic species in the same urine culture medium.

### Data collection and definitions of variables

To identify predictors of urinary tract infections caused by ESBL-E, we referred to previously reported studies on risk factors related to multidrug resistance, including ESBL. Demographic and clinical data were obtained from medical records. The collected variables included age, gender, comorbid diseases, hospital admission history, undergoing an invasive urological procedure (such as intubation or catheterization), treatment history, and antibiotic use in the past 3 months. Comorbid diseases included chronic diabetes mellitus, chronic renal failure, immunodeficiency, neoplasia, recurrent UTIs, and severe underlying disease. Hospital admission history included such items as admission times, hospital stays in preceding 3 months, and admission history to the medical department, surgical department, or ICU.

### Susceptibility testing

The drug susceptibility test used the paper diffusion method in accordance with the Clinical Laboratory Standards Institute (CLSI). The minimum inhibitory concentration (MIC) was passed through the Vitek 2 automated microbial identification system (Vitek AMS; bioMerieux Vitek Systems Inc., Hazelwood, Missouri). All results met the CLSI Enterobacteriaceae standards. Six types of antibacterial agents were tested: β-lactam/β-lactam Enzyme inhibitor combination (cefoperazone-sulbactam, piperacillin-tazobactam), cephalexin (ceftazolin, cefotaxime, ceftazidime, ceftriaxone, cefepime), carbapenem (imipenem, meropenem), aminoglycoside (amikacin), folate pathway inhibitor (trimethoprim-sulfamethoxazole), and fluoroquinolone (Levofloxacin) (Sigma-Aldrich, St. Louis, Missouri). Quality control was performed on *E. coli* (ATCC 25922) and *K. pneumoniae* (ATCC 700603) [12].

### Statistical analysis

As age and admission times are continuous variables with non-normal distribution, both were grouped into categories (0–18 years, 18–60 years, 60+ years); thus, all data existed in the form of categorical variables. We first numbered 874 patients, randomly sampled the numbers with the “sample” command in R software, and used the “set. Seed” and ind <-sample (n, 0.7 * n) commands at the same time. The randomly sampled patients were divided into a training set and a validation set. 611 patients (70% of the study) were randomly selected as the training set, and 263 patients (the other 30%) were selected as the validation set. Internal verification was carried out using a resampling-based method. Pearson's chi-square test or Fisher's exact test was used to compare differences between data sets, as appropriate, and for univariate analysis in the training set. All variables with a *P* value less than 0.1 in the univariate analysis were input into the multivariate analysis to further select the variables in the predictive model.

A predictive model was established by applying multivariate logistic regression with variables selected from multivariate analysis. The risk predictive model of ESBL-E infection was presented using a nomogram. The predictive model was evaluated on three criteria: discriminatory capacity, calibration ability, and clinical effectiveness. The AuROC was used to evaluate discriminative ability. The calibration curve and Hosmer–Lemeshow test were used to evaluate its calibration ability. Decision curve analysis (DCA) was used to evaluate clinical efficacy. All tests were two-tailed, and a P value of less than 0.05 was considered statistically significant. All statistical analyses were performed using R software (version 3.6.3, Vienna, Austria).

## Results

### Demographics and clinical characteristics

**Fig. 1:**
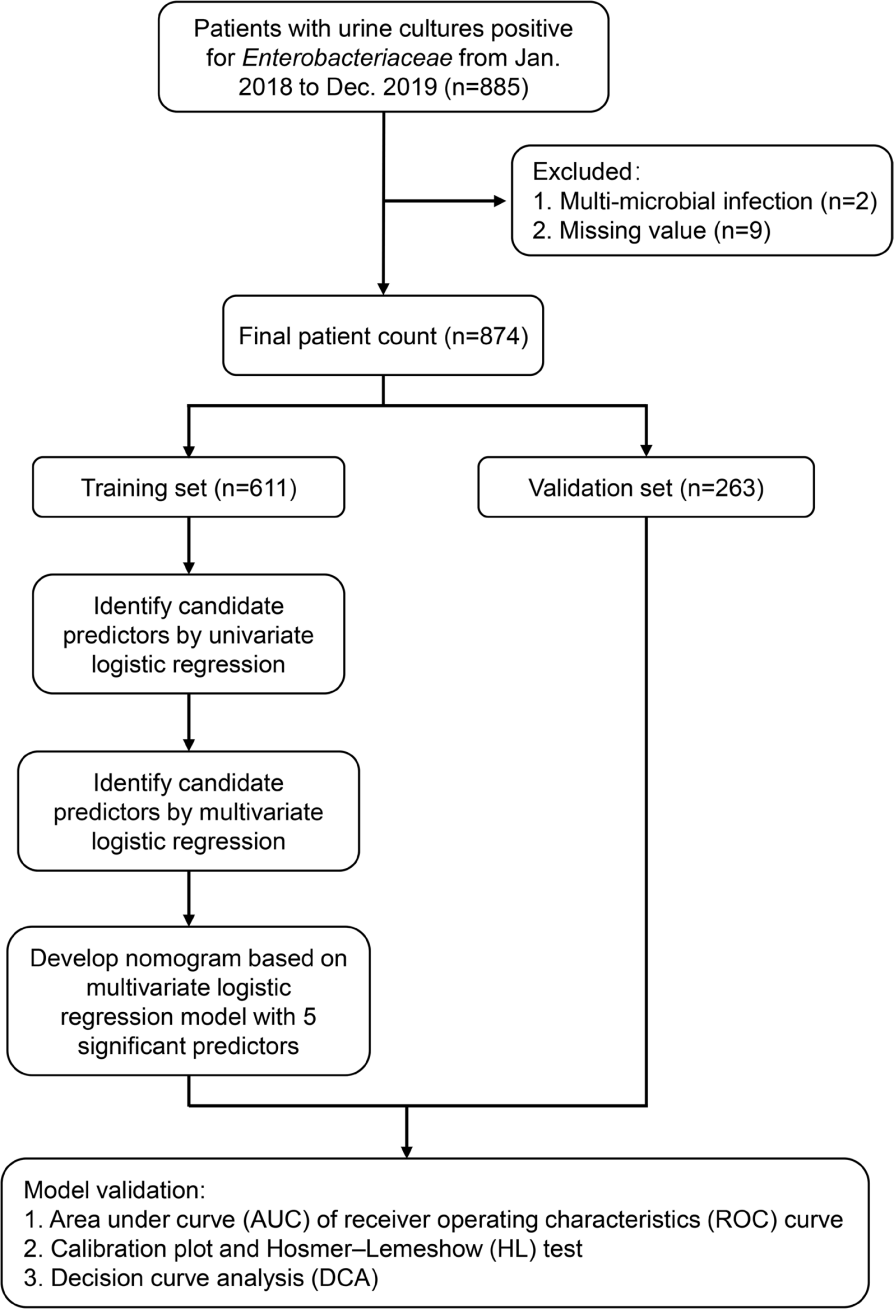
Experimental flowchart

Figure 1 shows the overall experimental flow. The organisms of interest, *E. coli* and *K. pneumoniae*, were identified in urine cultures from 885 unique patients during the study period. Of these, 11 were removed from the dataset: 9 cases were missing data, and 2 cases were identified to contain 2 pathogenic species. Table 1 shows a comparison of the demographic and clinical factors between the ESBL-E and non-ESBL-E patients. The median (IQR) ages at presentation were 65.5 (52–76) years for the ESBL-E group and 61 (49–74) years for the non-ESBL-E group (*P* < 0.001). The proportions of ESBL-E infections among males and females were 33.5% and 66.5%, respectively (*P* = 0.05). The two groups were compared across numerous factors: several comorbid diseases, hospital admission history, invasive urological procedure treatment history, and antibiotic use in the past 3 months. Those which showed significant differences (*P* < 0.05) were: diabetes mellitus, severe underlying disease, a hospital stay in the preceding 3 months, prior admission to the medical department, prior admission to the surgical department, prior admission to the ICU, undergoing an invasive urological procedure, and antibiotic use in the past 3 months. Among microorganisms, *E. coli* (739 cases, 84.6%) was the most commonly isolated species, with *K. pneumoniae* (135 cases, 15.4%) comprising the remainder.

**Table 1:** Demographic data, clinical characteristics

Variables	Overall (n = 874)	Non-ESBL-E (n = 602)	ESBL-E (n = 272)	*P* value
Gender, n (%)				0.050
Male	236 (27.0)	145 (24.1)	91 (33.5)	
Female	638 (73.0)	457 (75.9)	181 (66.5)	
Age (years), [median (IQR)]	62.0 (50–75)	61 (49–74)	65.5(52–76)	< 0.001
Comorbidity diseases				
Diabetes mellitus, n (%)				0.038
Yes	287 (32.8)	211 (35.0)	76 (27.9)	
No	587 (67.2)	391 (65.0)	196 (72.1)	
Chronic renal failure, n (%)				0.208
Yes	123 (14.1)	91 (15.1)	32 (11.8)	
No	751 (85.9)	511 (84.9)	240 (88.2)	
Immunodeficiency, n (%)				0.056
Yes	51 (5.8)	29 (4.8)	22 (8.1)	
No	823 (94.2)	573 (95.2)	250 (91.9)	
Neoplasia, n (%)				0.072
Yes	103 (11.8)	63 (10.5)	40 (14.7)	
No	771 (88.2)	539 (89.5)	232 (85.3)	
Recurrent Urinary tract infections, n (%)				< 0.001
Yes	134 (15.3)	66 (11.0)	68 (25.0)	
No	740 (84.7)	536 (89.0)	204 (75.0)	
Severe underlying disease, n (%)				0.009
Yes	66 (7.6)	36 (6.0)	30 (11.0)	
No	808 (92.4)	566 (94.0)	242 (89.0)	
Hospital stays in preceding 3 months, n (%)				< 0.001
Yes	309 (35.4)	168 (27.9)	141 (51.8)	
No	565 (64.6)	434 (72.1)	131 (48.2)	
Previous hospitalization department				
Medical department, n (%)				0.005
Yes	265 (30.3)	165 (27.4)	100 (36.8)	
No	609 (69.7)	437 (72.6)	172 (63.2)	
Surgical department, n (%)				< 0.001
Yes	174 (19.9)	99 (16.4)	75 (27.6)	
No	700 (80.1)	503 (83.6)	197 (72.4)	
Intensive Care Unit (ICU), n (%)				0.047
Yes	10 (1.1)	4 (0.7)	6 (2.2)	
No	864 (98.9)	598 (99.3)	266 (97.8)	
Invasive urological procedure, n (%)				< 0.001
Yes	269 (30.8)	150 (24.9)	119 (43.8)	
No	605 (69.2)	452 (75.1)	153 (56.3)	
Antibiotic use in the past 3 months, n (%)				< 0.001
Yes	190 (21.7)	91 (15.1)	99 (36.4)	
No	684 (78.3)	511 (84.9)	173 (63.6)	
Microorganism, n (%)				
*Escherichia coli*	739 (84.6)	540 (89.7)	199 (73.2)	< 0.001
*Klebsiella sp.*	135 (15.4)	62 (10.3)	73 (26.8)	< 0.001
Mortality, n (%)				
Secondary to infection	6 (0.7)	4 (0.7)	2 (0.7)	0.604
Other cause	7 (0.8)	3 (0.5)	4 (1.5)	0.140

Following random sampling, 611 patients, including 191 (31.3%) ESBL-E patients, were included in the training set. The remaining 263 patients, with 82 (31.2%) ESBL-E patients, were assigned to the validation set. No significant difference in the variables was observed between the training validation sets (all *P* > 0.05), as shown in Table 2.

**Table 2:** Clinical features and risk factor exposition in the study population

Variables	Overall (n = 874)	Training set (n = 611)	Validation set (n = 263)	*P* value
Status, n (%)				0.981
ESBL−	602 (68.9)	420 (68.7)	181 (68.8)	
ESBL+	272 (31.1)	191 (31.3)	82 (31.2)	
Gender, n (%)				0.111
Male	236 (27.0)	176 (28.8)	62 (23.6)	
Female	638 (73.0)	435 (71.2)	201 (76.4)	
Age, n (%)				0.543
0 to 18 years	70 (8.0)	49 (8.0)	21 (8.0)	
18 to 60 years	309 (35.4)	209 (34.2)	100 (38.0)	
Over 60 years	495 (56.6)	353 (57.8)	142 (54.0)	
Comorbidity diseases				
Diabetes mellitus, n (%)				0.920
Yes	287 (32.8)	200 (32.7)	87 (33.1)	
No	587 (67.2)	411 (67.3)	176 (66.9)	
Chronic renal failure, n (%)				0.998
Yes	123 (14.1)	86 (14.1)	37 (14.1)	
No	751 (85.9)	525 (85.9)	226 (85.9)	
Immunodeficiency, n (%)				0.913
Yes	51 (5.8)	36 (5.9)	15 (5.7)	
No	823 (94.2)	575 (94.1)	248 (94.3)	
Neoplasia, n(%)				0.170
Yes	103 (11.8)	78 (12.8)	25 (9.5)	
No	771 (88.2)	533 (87.2)	238 (90.5)	
Recurrent Urinary tract infections, n (%)				0.496
Yes	134 (15.3)	97 (15.9)	37 (14.1)	
No	740 (84.7)	514 (84.1)	226 (85.9)	
Severe underlying disease, n (%)				0.056
Yes	66 (7.6)	53 (8.7)	13 (4.9)	
No	808 (92.4)	558 (91.3)	250 (95.1)	
Hospital admission history				
Admission times, n (%)				0.555
1 to 2 times	645 (73.8)	455 (74.5)	190 (72.2)	
3 to 6 times	159 (18.2)	111 (18.2)	48 (18.3)	
More than 6 times	70 (8.0)	45 (7.4)	25 (9.5)	
Hospital stay in preceding 3 months, n (%)				0.645
Yes	309 (35.4)	219 (35.8)	90 (34.2)	
No	565 (64.6)	392 (64.2)	173 (65.8)	
Previous hospitalization department				
Medical department, n (%)				0.905
Yes	265 (30.3)	186 (30.4)	79 (30.0)	
No	609 (69.7)	425 (69.6)	184 (70.0)	
Surgical department, n (%)				0.111
Yes	174 (19.9)	113 (18.5)	61 (23.2)	
No	700 (80.1)	498 (81.5)	202 (76.8)	
Intensive Care Unit (ICU), n (%)				0.724
Yes	10 (1.1)	8 (1.3)	2 (0.8)	
No	864 (98.9)	603 (98.7)	261 (99.2)	
Treatment history				
Invasive urological procedure, n (%)				0.880
Yes	269 (30.8)	189 (30.9)	80 (30.4)	
No	605 (69.2)	422 (69.1)	183 (69.6)	
Antibiotic use in the past 3 months, n (%)				0.570
Yes	190 (21.7)	136 (22.3)	54 (20.5)	
No	684 (78.3)	475 (77.7)	209 (79.5)	

### Independent risk factors in the training set

The risk factor analysis was based on the 874 patients in the training set. Univariate and multivariate analysis for ESBL-E infection is shown in Table 3. Eleven variables were identified by univariate analysis (*P* < 0.1): gender, age, immunodeficiency, urinary tract infections, severe underlying disease, hospital stay in preceding 3 months, prior admission to medical department, prior admission to surgical department, prior admission to ICU, prior invasive urological procedure, and antibiotic use in the past 3 months.

**Table 3:** Univariate and Multivariate analysis in the training set

Variables	Univariate			Multivariate		
	OR	95% CI	*P* value	OR	95% CI	*P* value
Gender						
Male	1.654	1.143–2.388	0.0073	1.607	1.066–2.416	0.023
Female	Reference			Reference		
Age						
0 to 18 years	Reference			Reference		
18 to 60 years	3.712	1.529–11.108	0.008	2.825	1.119–8.679	0.043
Over 60 years	4.765	2.015–14.035	0.001	4.100	1.678–12.343	0.005
Diabetes mellitus						
Yes	0.884	0.610–1.274	0.513			
No	Reference					
Chronic renal failure						
Yes	0.886	0.529–1.446	0.637			
No	Reference					
Immunodeficiency						
Yes	1.829	0.914–3.606	0.082	1.671	0.770–3.579	0.187
No	Reference			Reference		
Neoplasia						
Yes	1.444	0.875–2.351	0.143			
No	Reference					
Recurrent urinary tract infections						
Yes	2.181	1.398–3.396	< 0.001	1.145	0.645–2.011	0.639
No	Reference			Reference		
Severe underlying disease						
Yes	2.294	1.295–4.058	0.004	1.536	0.805–2.907	0.188
No	Reference			Reference		
Admission times						
1 to 2 times	Reference					
3 to 6 times	1.047	0.664–1.627	0.841			
More than 6 times	1.380	0.719–2.580	0.320			
Hospital stay in preceding 3 months						
Yes	3.067	2.152–4.389	< 0.001	1.872	1.141–3.067	0.013
No	Reference			Reference		
Medical department						
Yes	1.516	1.052–2.179	0.025	0.799	0.498–1.266	0.344
No	Reference			Reference		
Surgical department						
Yes	1.751	1.145–2.663	0.009	0.943	0.572–1.533	0.816
No	Reference			Reference		
Intensive Care Unit (ICU)						
Yes	3.737	0.907–18.370	0.073	1.693	0.346–9.816	0.524
No	Reference			Reference		
Invasive urological procedure						
Yes	2.792	1.945–4.017	< 0.001	1.810	1.197–2.729	0.005
No	Reference			Reference		
Antibiotic use in the past 3 months						
Yes	3.231	2.179–4.807	< 0.001	1.833	1.055- 3.188	0.031
No	Reference			Reference		

Multivariate analysis was performed with the eleven variables identified by univariate analysis. Five variables were proved to be independent predictors for ESBL-E infection: male gender (OR = 1.607, 95% CI 1.066–2.416), older age (OR = 4.100, 95% CI 1.678–12.343), a hospital stay in preceding 3 months (OR = 1.872, 95% CI 1.141–3.067), invasive urological procedure (OR = 1.810, 95% CI 1.197–2.729), and antibiotic use within the previous 3 months (OR = 1.833, 95% CI 1.055–3.188).

### Predictive model construction and validation

**Fig. 2:**
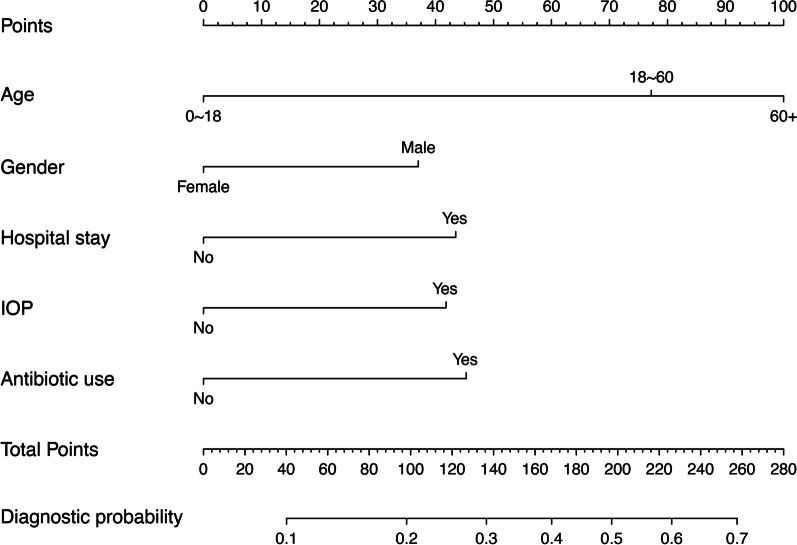
A nomogram for predicting the probability of ESBL-producing pathogen infection. Each variable is scored vertically against the Points scale at the top of the nomogram. The scores for all variables are then summed to obtain the patient’s Total Points, which are compared vertically against the corresponding Diagnostic possibility scale to estimate the probability of ESBL-producing pathogen infection

An ESBL-E infection risk estimation nomogram model was developed by logistic regression using the five independent predictors (Fig. 2). When present, each of the predictors contributes between 30 and 100 points to a final point total. This point total is then used to estimate the probability that the patient should can diagnosed as ESBL-E positive. To calculate the probability of a patient having ESBL-E infection, identify patient values on each axis, then for each draw a vertical line upwards to the “Points” axis. This determines how many points each variable generates. Add the points for all variables and locate this sum on the “Total points” line. Then draw a vertical line downwards from this point and identify the recurrence risk probability of ESBL-E infection. Hospital stay = Hospital stay in preceding 3 months; IOP = invasive urological procedure; Antibiotic use = Antibiotic use in the past 3 months.

**Fig. 3:**
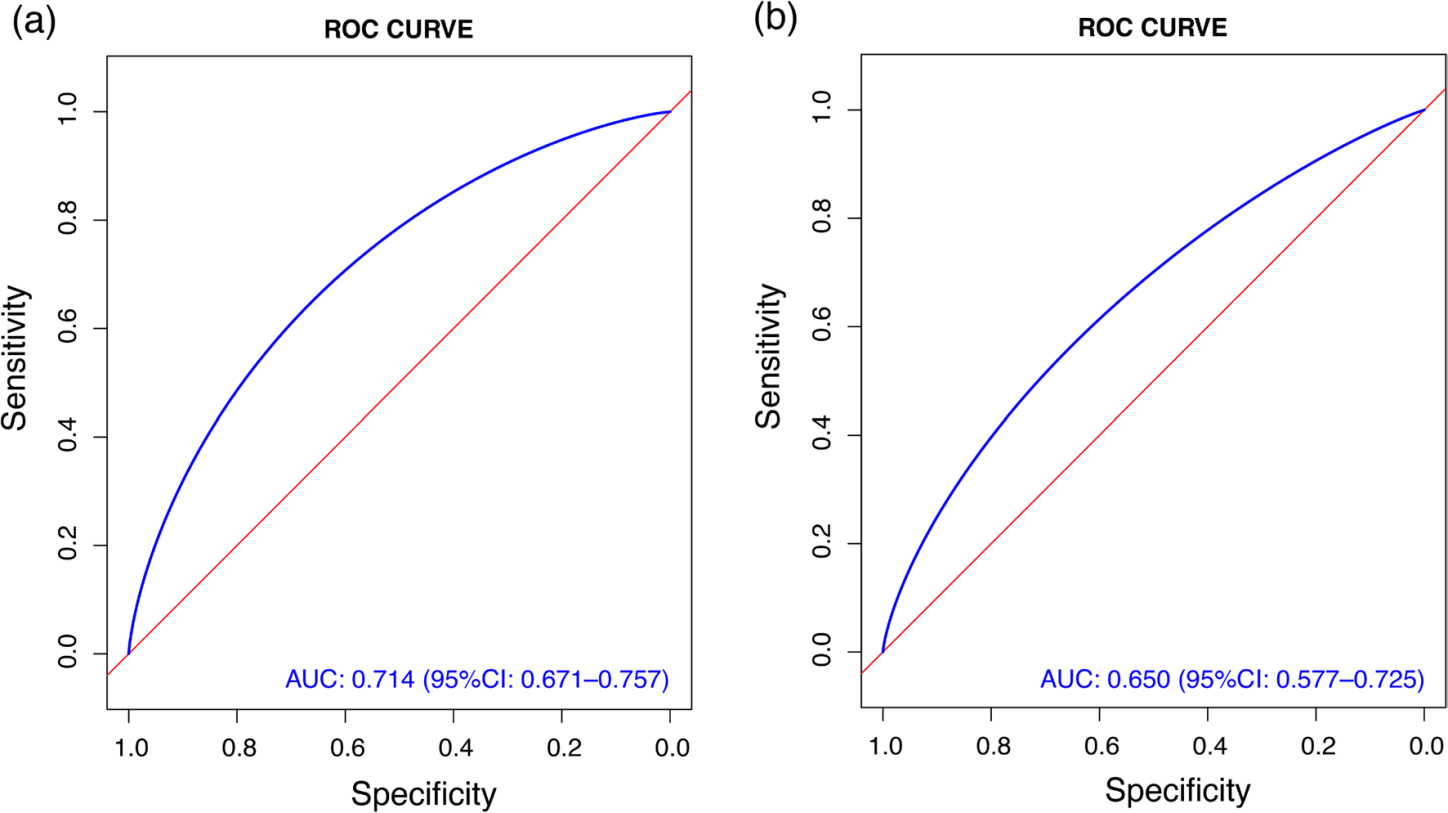
ROC curves form the training (**A**) and validation set (**B**) with AUC and 95% CI

The AUC was used to evaluate the discriminatory capacity of the predictive model, and the nomogram demonstrated good accuracy in estimating the risk of ESBL-E infection. The AUC of ROC was 0.714 (95% CI 0.671–0.757) in the training set (Fig. 3A). In validation set, the AUC of ROC was 0.650 (95% CI 0.577–0.725) (Fig. 3B).

**Fig. 4:**
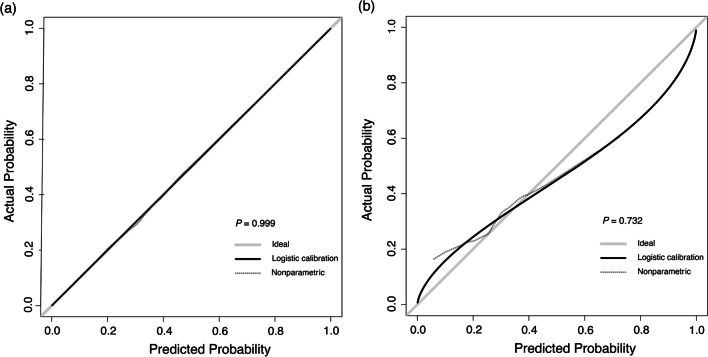
Calibration plots. The shadow line represents perfect prediction by an ideal model. The solid line shows the model’s performance using the training set (**A**) and validation set (**B**), with Hosmer–Lemeshow test *P* values of 0.999 and 0.732, respectively. The dotted line represents the predictive performance by a nonparametric model using the training set (**A**) and validation set (**B**)

**Fig. 5:**
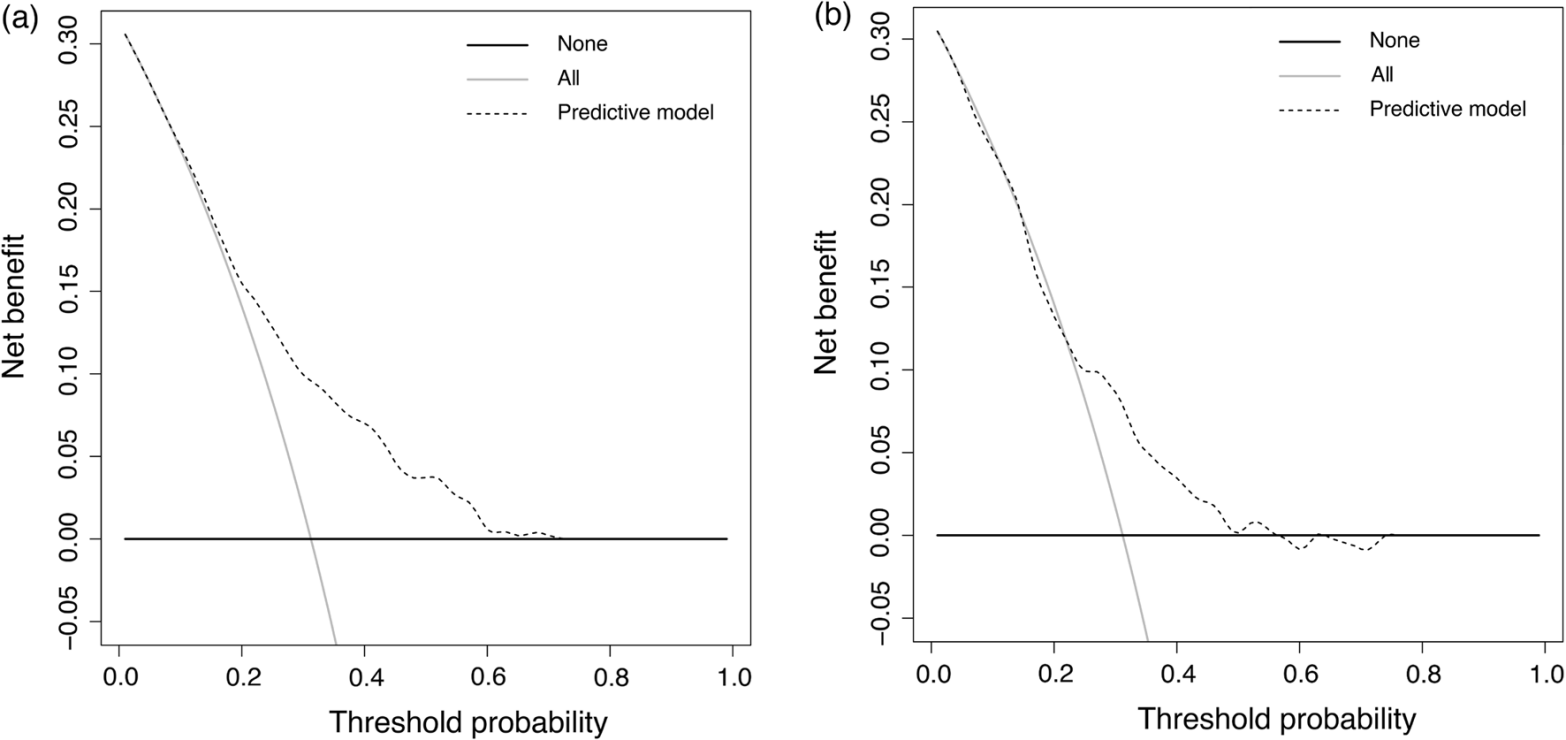
Decision curve analysis of the training set (**A**) and validation set (**B**). The black solid line indicates that no patients were treated. The grey solid line indicates that all patients were treated. The dotted line indicates treatment according to the model. The area between the dotted line, the grey solid line, and the black solid line represents the net benefit accrued by utilizing the model

A calibration plot and Hosmer–Lemeshow test were used to the calibrate the predictive model (Fig. 4). The calibration curves show the predictive model and the validation set produce very good fits of the data. The Hosmer–Lemeshow test indicates that the predicted probability is highly consistent with the actual probability (training set, *P* = 0.999; validation set, *P* = 0.732). Decision curve analysis, shown in Fig. 5, was used to demonstrate the net benefits of this predictive model. Its strong predictive capacity allows for accurate diagnosis, which should result in better patient treatment than either non-diagnosis or full diagnosis.

### Antibiotic susceptibility testing

Table 4 indicates the overall antimicrobial susceptibility of PE to the antibiotics tested. The highest sensitivity was observed with amikacin (94.7%), carbapenem (95.0%), polymyxin (99.2%), tigecycline (98.9%), and latamoxef (91.2%). Except for latamoxef and cefdinir, there are statistically significant (*P* < 0.05) differences in the susceptibility of all antibacterial drugs between the two groups.

**Table 4:** Antibiogram result of PE

Antibiotics	Antibiogram result								Total	SEN	*P* value
	Non-ESBL-E				ESBL-E						
	S	I	R	SEN	S	I	R	SEN			
Ciprofloxacin	304	24	265	51.3	39	5	220	14.8	857	40.0	< 0.001
Levofloxacin	246	66	246	44.1	35	18	219	12.9	874	32.2	< 0.001
P/T	580	14	8	96.3	167	35	69	61.6	873	85.6	< 0.001
Ceftazidime	540	43	16	90.2	26	49	196	9.6	870	65.1	< 0.001
C/S	590	7	3	98.3	166	31	75	61.0	872	86.7	< 0.001
Cefepime	533	23	45	88.7	18	46	207	6.6	872	63.2	< 0.001
Aztreonam	561	1	30	94.8	18	2	242	6.9	854	67.8	< 0.001
Amikacin	595	4	3	98.8	233	4	35	85.7	874	94.7	< 0.001
Tobramycin	438	115	39	74.0	127	53	81	48.7	853	66.2	< 0.001
Carbapenem	602	0	0	100.0	228	0	44	83.8	874	95.0	< 0.001
TMP-SMX	312	1	288	51.9	82	0	188	30.4	871	45.2	< 0.001
Polymyxin	600	0	2	99.7	267	0	5	98.2	874	99.2	0.033
Doxycycline	271	123	204	45.3	73	43	155	26.9	869	39.6	< 0.001
Minocycline	371	80	139	62.9	96	39	126	36.8	851	54.9	< 0.001
Tigecycline	590	0	2	99.7	238	0	7	97.1	837	98.9	0.004
Cefixime	45	0	134	25.1	1	0	235	0.4	415	11.1	< 0.001
Latamoxef	53	0	2	96.4	50	0	8	86.2	113	91.2	0.095
Cefdinir	1	0	20	4.8	1	0	93	1.1	115	1.7	0.333
Ceftriaxone	388	0	3	99.2	2	0	45	4.3	438	89.0	< 0.001
Cefmetazole	92	0	0	100.0	4	0	14	22.2	110	87.3	< 0.001
Ceftizoxime	39	0	0	100.0	0	0	10	0.0	49	79.6	< 0.001

S, Sensitive; I, Intermediate; R, Resistant; SEN, Sensitivity, %; P/T, piperacillin/tazobactam; C/S, cefoperazone/sulbactam; TMP-SMX, trimethoprim–sulfamethoxazole

## Discussion

In this study, we propose a valuable clinical tool to predict the possibility of ESBL producing organisms as the etiology of urinary tract infection in hospitalized patients. Our prediction tool consists of five variables, including three clinical categories: gender, age, hospital stay in the preceding 3 months, invasive urological procedures, and antibiotic use in the past 3 months. The scoring system is feasible in clinical practice because it contains patient factors that can be easily determined from medical records and can be implemented with minimal health system cost.

First, we found that older patients were significantly more likely to get ESBL-E infections. Older age more likely to get ESBL-E infections, which is in agreement with prior studies [13, 14]. Second, in univariate and multivariate regression analysis, gender specifically, being male is an independent risk factor. Many previous studies similarly consider being male a predictor of infection [10, 15]. Third, we showed that prior hospital stays were a predictor for ESBL-E infection. This comports well with previous work which shows that hospital stays increase the risk of carrying ESBL-E [16]. The epidemiology of these ESBL-producing bacteria is becoming more and more complicated [17]. Fourth, we included invasive urological procedures, such as intubation and catheterization, as an ESBL-E UTI predictor, in agreement with previously published literature [18]. Invasive procedures can damage the skin and mucous membranes, thereby increasing the chance of contact with ESBL-producing bacterial strains [19]. Lastly, in this study, we found an association between the use of antibiotics in the past 3 months and the occurrence of ESBL-E in UTI. The abuse of antibiotics in recent years has led to an increase in antibiotic resistance. ESBL-E colonization is a known risk factor for subsequent infection or bacteremia [20, 21]. Additionally, the improper use of antibacterial drugs has been shown to play a key role in the emergence of multi-drug resistant organisms. The selection of resistant forms may occur during or after antimicrobial treatment.

Further, comorbidities such as diabetes, chronic renal insufficiency, serious underlying diseases, and tumors were not considered predictors of UTIs caused by ESBL-E [22], and they were found to not be significant contributors in this study either.

Nomogram is based on multi-factor regression analysis, integrating multiple predictive indicators, and then using scaled line segments, drawn on the same plane according to a certain ratio, so as to express the relationship between the various variables in the prediction model mutual relations. The nomogram transforms the complex regression equation into a visualized graph, making the results of the prediction model more readable and facilitating the evaluation of the patient [23].

The production and evaluation of nomogram is in accordance with conventional methods. The ROC curves, Decision curve analysis and Calibration plots in the article are all evaluations of nomogram, indicating that our nomogram is reasonable. E.g., Assuming a patient of 65 age, male, who has no history of hospitalization, has not undergone surgery but has used antibiotics within three months. We calculate the total points are 160, then the probability of an ESBL-E infection is 38%. Our clinical prediction tool provides doctors with an easy-to-use mechanism to improve the method of determining which patients with urinary tract infection may need broad-spectrum antibiotic coverage. The purpose of wider spectrum coverage is to ensure that individuals are initially treated with appropriate antibiotics. The tool showed excellent discrimination in the derivation queue with AUC of 0.714, but only moderate discrimination in the verification queue with AUC of 0.650. Because urine samples are highly contaminated clinical biological samples, considering the high risk of urine contamination, the AUC of our prediction model is acceptable. Although our nomogram is not perfect and could not be 100% predicted, when patients find urinary tract infection, it has certain guiding significance for doctors' initial medication decision, and more reasonable antibiotics can be selected as soon as possible.

In addition to building a predictive model, numerous carbapenem antibiotics were tested against ESBL-E cultures to determine whether these resistant bacteria could be combatted by less-common treatments. A previous study found that there was a correlation between CTX-M-producing bacteria, one of the three most common ESBLs genes in *E. coli* and *K. pneumoniae*, and fluoroquinolones resistance [24]. They showed that ESBL-E had an 87.1% resistance rate to levofloxacin. It has been demonstrated that antibiotics (prophylactic or therapeutic) can induce antibiotic resistance genes that respond to ESBL-E infection [25]. Nonstandard antibiotic treatments, such as those explored here, are therefore necessary.

We showed that carbapenems and aminoglycosides, such as amikacin, seem to be good choices for the treatment of serious infectious diseases of ESBL-E, though they may introduce other complicating factors such as the need to closely monitor renal response. Previous studies have shown that the proportion of carbapenem-resistant producing-Enterobacteriaceae in UTIs is less than 3% [26–28]. However, in this study, we found that carbapenem-resistant producing-Enterobacteriaceae could be as high as 5%, especially in the ESLB-E group, the resistance rate of carbapenems was 16.2%.

Tigecycline and polymyxin were also demonstrated to be highly effective against ESBL-E. Previous work has found that tigecycline has clinical effectiveness in the treatment of UTIs; however, its use is still controversial due to a lack of data and randomized controlled trials [29]. The authors recommended using tigecycline only in the absence of other potential treatments; if aminoglycosides or β-lactams can be used to treat UTI, tigecycline should be avoided. Similarly, while polymyxin is shown to be an effective treatment for UTIs, its partial conversion to colistin in the urine may induce nephrotoxicity, so it should be used with caution.

The effectiveness of piperacillin/tazobactam and cefoperazone/sulbactam against ESBL-E were about 60%. ESBLs are generally inhibited by tazobactam [18], which could be a suitable option for initial empirical medication of ESBL-E high-risk groups. Latamoxef showed high effectiveness against ESBL-E, but due to the small number of subjects using the drug, further verification is needed.

This study has several limitations and ways it could be improved in the future. First, it is a retrospective case–control study with election bias. Second, some data may be missing from the medical records. Third, this study was conducted in a large hospital in China, and only inpatients were recruited; therefore, patients may not be representative of the greater Chinese or world populations. Finally, the overall sample size was too small to include more research factors, but the age span of the population included in this study was large. In the next step of the research, we will increase the sample size, more stringent review of electronic medical records, sub-group analysis for different age groups, and external verification to optimize the model to improve prediction accuracy. Since the proportion of ESBL + resistant strains in our hospital have reached more than 30%, it is very valuable to find a clinical prediction model that could improve the drug resistance of urinary tract infections. Our prediction model is more suitable for hospitals with high ESBL + resistance rates as a reference.

## Conclusion

The prevalence of ESBL-E in patients with urinary tract infections in the Chinese hospital system continues to grow, especially among men and the elderly. Hospitalization in the first 3 months, invasive urological procedures, and the use of antibiotics in the past 3 months further increase the risk of infection. The nomogram developed in this study can be used to identify high-risk patients. These patients may benefit from empirical antibiotic prescriptions, such as those explored in this study. Doing so may reduce the failure rate of treatment as promote responsible use of antibiotics which might otherwise contribute to the growing trend of antibiotic resistance.

## Data Availability

The datasets used and/or analysed during the current study are available from the corresponding author on reasonable request.
